# Homocysteine Drives Hippocampal Blood–Brain Barrier Disruption and Cognitive Decline Under Chronic Stress via DNA Hypomethylation of Cav1.2

**DOI:** 10.3390/brainsci16050491

**Published:** 2026-04-30

**Authors:** Mao-Yang Zhou, Jin-Shan Li, Zhao-Xin Sun, Jie Yin, Yun Zhao, Fang Xie, Xue Wang, Sheng-Hui Zhang, Zhao-Wei Sun, Ling-Jia Qian

**Affiliations:** Department of Neurobiology, Beijing Institute of Basic Medical Sciences, Beijing 100850, China; z18355454174@126.com (M.-Y.Z.); lj_shan99@163.com (J.-S.L.); 202110200019@stu.tjus.edu.cn (Z.-X.S.); bz3209086@126.com (J.Y.); zhaoyun@bmi.ac.cn (Y.Z.); xiefang@bmi.ac.cn (F.X.); wangxue@bmi.ac.cn (X.W.); 1701111498@pku.edu.cn (S.-H.Z.); sunzhaowei@bmi.ac.cn (Z.-W.S.)

**Keywords:** chronic stress, Hcy, hippocampal BBB, BMECs, Cav1.2, DNA hypomethylation

## Abstract

**Highlights:**

**What are the main findings?**

- Chronic stress induces homocysteine (Hcy) accumulation, which directly impairs the barrier-forming function of hippocampal brain microvascular endothelial cells (BMECs).
- Elevated Hcy triggers blood–brain barrier (BBB) breakdown by inducing DNA hypomethylation of Cacna1c, leading to Cav1.2 overexpression, reactive oxygen species (ROS) burst, and tight junction degradation.

**What are the implications of the main findings?**

- This study establishes the Hcy-Cav1.2 metabolic–epigenetic axis as a novel pathological mechanism and therapeutic target for stress-related neurovascular damage.
- Lowering Hcy levels via accessible interventions, such as vitamin B supplementation, holds significant translational potential for rescuing BBB integrity and cognitive function under chronic stress.

**Abstract:**

**Background:** Chronic stress is a major risk factor for cognitive decline and blood–brain barrier (BBB) disruption, yet the underlying molecular mechanisms remain elusive. This study aimed to investigate the specific role of the metabolic intermediate homocysteine (Hcy) in chronic stress-induced BBB dysfunction and cognitive impairment. **Methods:** We utilized a male Sprague-Dawley rat model of chronic unpredictable mild stress (CUMS) and administered vitamin B complex to lower Hcy levels in vivo. Regional Hcy accumulation, BBB permeability, and cognitive behaviors were assessed. In vitro, primary rat brain microvascular endothelial cells (BMECs) were exposed to Hcy to evaluate barrier-forming function, transcriptomic alterations, DNA methylation patterns, Cav1.2 expression, and reactive oxygen species (ROS) production. **Results:** CUMS selectively induced BBB hyperpermeability and significant Hcy accumulation predominantly within the rat hippocampus, which correlated intimately with cognitive deficits. Lowering Hcy levels via vitamin B supplementation successfully restored hippocampal BBB integrity and alleviated cognitive impairment. In addition, elevated Hcy severely impaired the barrier function of BMECs. Mechanistically, Hcy reduced global DNA methylation in BMECs and specifically induced targeted DNA hypomethylation at the intro region of *Cacna1c*. This epigenetic shift caused the transcriptional derepression and overexpression of the Cav1.2 calcium channel. Upregulated Cav1.2 subsequently triggered a robust ROS burst, leading to tight junction degradation. **Conclusions:** Our findings unveil a novel metabolic–epigenetic axis where Hcy-driven *Cacna1c* hypomethylation directly disrupts BMECs function to dismantle the hippocampal BBB. Lowering Hcy or targeting this Hcy-Cav1.2 pathway establishes a promising therapeutic strategy for mitigating stress-related neurovascular damage and cognitive disorders.

## 1. Introduction

Chronic stress is a non-specific adaptive response to environmental and social challenges. Persistent exposure to stress significantly elevates the risk of cognitive dysfunction across a spectrum of neurological disorders [1,2]. For instance, hyperactivation of the hypothalamic–pituitary–adrenal (HPA) axis—a hallmark of stress pathology—is consistently linked to cognitive and affective dysregulation in major depressive disorder (MDD) patients [3]. Epidemiological data further underscore that chronic psychosocial stress in adulthood is a potent risk factor for dementia and late-onset Alzheimer’s disease (AD) [4,5]. Nevertheless, the precise neurobiological mechanisms linking chronic stress to cognitive decline remain elusive, hindering the development of targeted interventions.

The blood–brain barrier (BBB), a specialized structure comprising brain microvascular endothelial cells (BMECs), pericytes, and astrocytic end-feet, is essential for maintaining central nervous system (CNS) homeostasis, and its impairment contributes critically to the progression of neurodegenerative diseases [6,7,8,9]. In AD, BBB breakdown facilitates amyloid-β (Aβ) accumulation and neuronal injury, while in Parkinson’s disease, cerebrovascular dysfunction is closely tied to neurodegeneration. Consistent with these observations, our previous studies have shown that stress-induced BBB disruption directly exacerbates cognitive deficits, identifying the neurovascular unit (NVU) as a promising therapeutic target [10,11]. However, the intrinsic molecular mechanisms through which stress induces barrier damage remain poorly understood.

Homocysteine (Hcy) is a well-established metabolic risk factor for cognitive impairment and BBB disruption [12,13,14]. For instance, hyperhomocysteinemia (HHcy) mice exhibit cognitive impairment accompanied by significant endothelium damage within the cerebral cortex and hippocampus [11,15]. In addition, Hcy treatment in human brain microvascular endothelial cells markedly downregulates the expression of tight junction-associated genes. However, it remains unclear how Hcy orchestrates these endothelial alterations under stress. As a precursor to S-adenosylhomocysteine (SAH), which is a potent endogenous inhibitor of methyltransferases, Hcy is biochemically positioned to disrupt gene-specific DNA methylation patterns [14]. These epigenetic alterations can, in turn, disrupt the transcriptional regulation of multiple BBB-related genes, including those encoding tight junction proteins [13,16,17]. Also, we previously demonstrated that Hcy-mediated DNA hypomethylation of the *Bdnf* gene contributes to stress-induced neuronal dysfunction and cognitive decline. Thus, Hcy-mediated epigenetic dysregulation likely serves as a central pathological switch driving neurovascular dysfunction.

Here, we demonstrate that exposure to chronic unpredictable mild stress (CUMS) significantly elevates circulating and hippocampal Hcy levels, which intimately correlate with BBB disruption and cognitive deficits. Moreover, transcriptome analysis identified the calcium channel Cav1.2 as a primary upregulated target of Hcy, the overactivation of which could trigger robust reactive oxygen species (ROS) burst and subsequent tight junction degradation. Furthermore, we discovered that Hcy reduced the global methylation potential of BMECs and inhibited DNA methylation of Cacna1c gene (encoding Cav1.2), thereby promoting its transcription. Collectively, our findings unveil a novel Hcy-Cav1.2 epigenetic axis in the regulation of BMECs function and BBB integrity, establishing a promising therapeutic target for stress-related cognitive disorders.

## 2. Materials and Methods

### 2.1. Animals

Adult male Sprague-Dawley rats (6 weeks old and weighing 120–140 g) purchased from Laboratory Animal Center of the Academy of Military Sciences (AMS) were randomly assigned to different groups using a computer-generated random number table. Rats were exposed to a 12 h/12 h light/dark cycle and had free access to pure water and food except during the stress intervention and behavioral experiments. All the experiments were approved by the Institutional Animal Care and Use Committee of the AMS (Permit No: IACUC-DWZX-2022-738) and were in accordance with the NIH Guide for the Care and Use of Laboratory Animals (NIH Publications No. 8023) and the ARRIVE guidelines from NC3Rs. We made all efforts to minimize animal suffering. All operators performing the behavioral tests and outcome assessment were blinded to group allocation.

### 2.2. CUMS Paradigm

This study employed the classic CUMS model to simulate the effects of chronic stress on rats. Previous studies have demonstrated that 8 weeks of CUMS exposure is sufficient to induce cognitive impairment, and substantial evidence has established that CUMS damages BBB integrity. Rats were randomly allocated to experimental groups using a computer-generated randomization protocol, with group assignment performed by an investigator independent of subsequent behavioral testing. In our experiment, rats in the CUMS group were exposed to one of the following chronic unpredictable mild stressors: restraint for 8 h; food and water deprivation for 24 h; cage tilt at 45° for 24 h; wet bedding for 24 h; forced swimming in 4 °C water for 3 min followed by towel drying; or continuous illumination for 24 h. These stressors were administered randomly each day over an 8-week period. To prevent habituation to any single stimulus, the same stressor was not applied on consecutive days. Non-stressed animals remained undisturbed in their home cages, except during routine cage cleaning procedures. For outcome assessment, a single-blind procedure was adopted: CUMS administration and behavioral testing were conducted by separate operators, and the assessors were blinded to group allocation during data acquisition and analysis.

### 2.3. Vitamin B Complex Administration

Our previous studies have demonstrated that vitamin B complex (comprising vitamin B6 (Solarbio, Beijing, China) at 24 mg/kg/day, vitamin B12 (Solarbio, Beijing, China) at 20 μg/kg/day, and folic acid (Sigma-Aldrich, St. Louis, MO, USA) at 10 mg/kg/day) reduces plasma Hcy levels and thereby improves cognitive function. During the 8-week stress period, rats in the homocysteine intervention group (CUMS + VBco) received daily intragastric administration of vitamin B complex at 09:00. The control intervention group underwent the same vitamin B complex gavage at the same time point. Both control and experimental groups received an equal volume (1.5 mL) of saline gavage at corresponding time points. All rats were acclimated to gentle handling and restraint prior to the experiment. To minimize stress responses during the procedure, a single skilled researcher performed all intragastric infusions.

### 2.4. Behavioral Tests

#### 2.4.1. Novel Object Recognition Test (NORT)

The apparatus consisted of a square open-field box (65 cm × 65 cm × 45 cm, Zhongshi, Beijing, China) with black-painted inner walls. The test consisted of three phases: habituation, familiarization, and test. One day before the familiarization phase, rats were habituated to the empty arena for 10 min. Twenty-four hours later, rats were placed in the same arena containing two identical white cubes (5 cm × 5 cm × 5 cm) and allowed to explore until they had accumulated 30 s of total object exploration time, or for a maximum of 5 min. One hour after the familiarization phase, one of the cubes was replaced with a novel object (a 5 cm-high aluminum can). During the 5 min test phase, the time spent exploring the novel object (T_<new>_) and the familiar object (T_<old>_) was recorded. Exploration was defined as directing the nose toward the object at a distance ≤2 cm, touching it with the nose or whiskers, or sniffing. The recognition index was calculated as follows: Recognition Index = T_<new>_/(T_<new>_ + T_<old>_).

#### 2.4.2. Morris Water Maze Test (MWMT)

The Morris water maze apparatus consisted of a circular pool (1.2 m in diameter, 75 cm in depth, Zhongshi, Beijing, China) with a black-painted bottom and walls. Distinctive visual cues of various shapes and colors were placed on the walls and surrounding curtains to serve as spatial references. Water temperature was maintained at 24 ± 1 °C. Rats were trained over five consecutive days to locate a hidden platform (2 cm below the water surface). Each daily session consisted of one trial lasting a maximum of 3 min. Escape latency and swim trajectories were recorded. If a rat failed to locate the platform within 3 min, it was gently guided to the platform and allowed to remain there for at least 10 s. A probe test was conducted 24 h after the final training session, during which the platform was removed and rats were allowed to swim freely for 3 min. Platform crossing frequency and time spent in the target quadrant were recorded during the probe test to assess spatial memory.

### 2.5. Transmission Electron Microscopy (TEM)

Rat hippocampal tissues were dissected on ice-cold plates and immediately immersed in 2.5% glutaraldehyde (Solarbio, Beijing, China) at 4 °C for 2 h for primary fixation. Samples were then washed with 0.1 M phosphate buffer (pH 7.4, Solarbio, Beijing, China), post-fixed with 1% osmium tetroxide in 0.1 M sodium cacodylate buffer (pH 7.4, Sinopharm, Shanghai, China) for 1–2 h, dehydrated through a graded ethanol series followed by propylene oxide (Sinopharm, Shanghai, China), and embedded in resin (Sinopharm, Shanghai, China), which was then polymerized at 60 °C for 48 h. Ultrathin sections (60–80 nm) were cut, stained with uranyl acetate and lead citrate (Aladdin, Shanghai, China), and imaged using a transmission electron microscope.

### 2.6. Quantitative Real-Time PCR (qRT-PCR)

Total RNA was extracted from cells or tissues using TRIzol reagent (catalog number 93289, Sigma-Aldrich, St. Louis, MO, USA). Subsequently, 2 μg of RNA was reverse transcribed into cDNA using RT Master Mix (catalog number G490, Abmart, Shanghai, China). Target gene expression was quantified by SYBR Green-based qPCR on a LightCycler 96 Real-Time PCR System (Roche, Rotkreuz, Switzerland). Relative expression levels were calculated using the 2^−ΔΔCt^ method. Primer sequences are listed in Table S1.

### 2.7. Western Blotting

Dissected rat hippocampal tissues were lysed in RIPA buffer (Applygen, Dhaka, Bangladesh, C1053+) supplemented with protease inhibitor cocktail (Mce, Singapore, HY-K0010) at 1 mL per 100 mg of tissue, and homogenized using an ultrasonic homogenizer. After lysis on a shaker at 4 °C for 30 min, the lysates were centrifuged at 12,000 rpm for 15 min at 4 °C to collect the supernatants. Protein concentrations were determined using the bicinchoninic acid (BCA) assay.

Protein samples (20 μg) were loaded onto 10% SDS-polyacrylamide gels without heat treatment, resolved by electrophoresis, and transferred onto PVDF membranes via wet transfer. Membranes were blocked with 5% (*w*/*v*) non-fat milk in TBST for 1.5 h at room temperature, followed by overnight incubation at 4 °C with primary antibodies against the following targets: Claudin-5 (1:1000; Thermo Fisher Scientific, Waltham, MA, USA, #35-2500), Occludin (1:1000; Mce, Monmouth Junction, NJ, USA, HY-P81155), ZO-1 (1:1000; Invitrogen, Carlsbad, CA, USA, 61-7300) and β-actin (1:20,000; Abclonal, Wuhan, China, AC038).

After three 6 min washes with TBST, the membranes were incubated for 2 h at room temperature with the following secondary antibodies: goat anti-mouse IgG (H + L) (1:5000; ZSGB-BIO, Beijing, China, #ZB-5305) and goat anti-rabbit IgG (H + L) (1:5000; ZSGB-BIO, #ZB-5301). Following five additional 6 min washes with TBST, protein bands were visualized using enhanced chemiluminescence (ECL), and band intensity was quantified using ImageJ software (version 1.54f, National Institutes of Health, Bethesda, MD, USA).

### 2.8. Blood–Brain Barrier Permeability Assessment

BBB permeability was assessed using a sodium fluorescein (NaFl) method following previously established protocols. Under gentle restraint, NaFl (20 mg/mL, 5 mL/kg) was slowly injected via the tail vein using a 30° angled venous needle. After 50 min of circulation, rats were anesthetized with pentobarbital sodium (80 mg/kg, i.p.), and serum samples were collected. Rats were then transcardially perfused with 0.9% saline, and the hippocampus, prefrontal cortex, striatum, and hypothalamus were dissected and weighed. Tissue samples were homogenized, diluted 1:1 (*v*/*v*) with 2% trichloroacetic acid, incubated overnight at 4 °C, and then centrifuged at 3000 rpm for 10 min at 4 °C. The resulting supernatants were diluted 1:1 (*v*/*v*) with 0.05 M borate buffer (pH 9.0). Standards and samples were loaded into 96-well plates, and fluorescence intensity was measured (NaFl: excitation 480 nm, emission 538 nm). BBB permeability was expressed as the brain-to-serum fluorescence intensity ratio, normalized to control group values, and presented as fold change.

### 2.9. Enzyme-Linked Immunosorbent Assay (ELISA)

Blood samples were allowed to clot at room temperature for 1 h, then centrifuged at 1000 rpm for 15 min to obtain serum. Following anesthesia, hippocampus, prefrontal cortex, striatum, and hypothalamus were dissected, diluted in phosphate-buffered saline (PBS) (1 mL per 100 mg), and homogenized using an ultrasonic homogenizer. Homogenates were diluted 1:4, and Hcy levels in serum and tissues were measured using an Hcy ELISA kit (CUSABIO, Houston, TX, USA, CSB-E08896r) according to the manufacturer’s instructions.

For global DNA methylation analysis, tissues were processed as above. DNA was extracted using a DNA extraction kit (Vazyme, Nanjing, China, DC112), and concentration and purity were assessed by spectrophotometry to ensure consistency across samples. Global methylation levels were determined using a global DNA methylation assay kit (Epigentek, Farmingdale, NY, USA, P-1030-96) following the manufacturer’s protocol.

### 2.10. Cell Culture and Treatments

Primary rat brain microvascular endothelial cells (rBMECs) were isolated from the cerebral forebrain of 18–22-day-old SD rats using a rat brain microvascular endothelial cell isolation kit (Procell, Bethel, CT, USA, P-CA-601) following the manufacturer’s instructions. Briefly, the cerebral forebrain was dissected and minced, then digested with the provided specialized digestive solutions. The tissue homogenate was filtered through a 100 μm cell filter, and the isolated microvessels were collected by centrifugation using the specialized isolation solution. The obtained cells were resuspended in planting solution and seeded into T25 culture flasks. After 48 h of culture, the cells were purified using the screening solution to eliminate contaminating cells. Cells were cultured at 37 °C in a humidified atmosphere containing 5% CO_2_ and subjected to interventions and subsequent experiments after one passage. rBMECs were maintained in complete medium containing endothelial cell growth supplements (Procell, Canton, MA, USA, CM-R108).

A pre-prepared 50 μM Hcy solution (Sigma-Aldrich, H4628) dissolved in ddH_2_O was used for interventions, with ddH_2_O serving as the vehicle control. Cells were harvested and stored for subsequent analyses.

### 2.11. Cell Functional Assays

#### 2.11.1. Cell Proliferation Assay

Cell proliferation was assessed using the Cell Counting Kit-8 (CCK-8) assay (Mce, Monmouth Junction, NJ, USA). rBMECs were seeded in 96-well plates at a density of 5 × 10^3^ cells per well. After the indicated interventions, 10 μL of CCK-8 solution was added to each well, and the plates were incubated at 37 °C for an additional 2 h. Absorbance at 450 nm was measured using a microplate reader, with blank medium used as a reference. Cell proliferation rates were calculated relative to the control group and expressed as a percentage of control.

#### 2.11.2. Cell Apoptosis Assay

Apoptosis was detected using Annexin V-FITC/PI double staining followed by flow cytometry. rBMECs including those detached in the culture supernatant were collected, washed twice with ice-cold PBS, and resuspended in 1× binding buffer. Annexin V-FITC and PI staining were performed according to the manufacturer’s instructions (Mce, Monmouth Junction, NJ, USA), followed by incubation at room temperature in the dark for 15 min. Flow cytometric analysis was conducted within 1 h to quantify the percentages of early apoptotic (Annexin V^+^/PI^−^) and late apoptotic/necrotic (Annexin V^+^/PI^+^) cells.

#### 2.11.3. Cell Migration Assays

Wound Healing Assay. Collective cell migration was assessed using the scratch assay. rBMECs were seeded in 6-well plates and cultured until they reached >90% confluence. A straight scratch was made vertically across the center of each well using a sterile 200 μL pipette tip. Detached cells were gently removed by washing three times with PBS. The medium was then replaced with medium containing 1% fetal bovine serum (FBS) supplemented with the respective interventions. Images of the same scratch position were captured immediately (0 h) and 24 h post-intervention using an inverted microscope. Scratch width was measured using ImageJ software (National Institutes of Health, USA), and the migration rate was calculated as follows: Migration rate (%) = [(W_0h_ − W_24h_)/W_0h_] × 100%, where W represents the scratch width.

Transwell Migration Assay. Directional cell migration was assessed using Transwell chambers with 8.0 μm pore polycarbonate membranes. After the indicated interventions, rBMECs were harvested, resuspended in serum-free medium, and counted. A 100 μL aliquot of cell suspension containing 5 × 10^4^ cells was seeded into the upper chamber, while 600 μL of complete medium containing 20% FBS (as a chemoattractant) was added to the lower chamber. The chambers were incubated at 37 °C in a humidified atmosphere containing 5% CO_2_ for 24 h. Non-migrated cells remaining on the upper surface of the membrane were gently removed with a cotton swab. The membranes were then fixed in 4% paraformaldehyde for 15 min and stained with 0.1% crystal violet for 20 min. After gently washing with PBS, cells on the lower surface of the membrane were imaged in at least five random fields at 200× magnification using an inverted microscope. The number of migrated cells was counted, and the average count per well was used for statistical analysis.

#### 2.11.4. Tube Formation Assay

In vitro angiogenic potential was assessed using a Matrigel tube formation assay. Matrigel (Corning, Corning, NY, USA, 356234) was thawed overnight at 4 °C, and 50 μL was added to each well of a 96-well plate, followed by polymerization at 37 °C for 30 min. After the indicated interventions, rBMECs were harvested and seeded at a density of 2 × 10^4^ cells per well onto the polymerized Matrigel. Cells were incubated at 37 °C in a humidified atmosphere containing 5% CO_2_ for 4–8 h. Tube-like structures were imaged using an inverted microscope, and total tube length, number of branch points, and number of mesh-like structures were quantified using ImageJ software (National Institutes of Health, USA).

#### 2.11.5. Barrier Function Assays

Transepithelial electrical resistance (TEER) Measurement. rBMECs were seeded at a high density (1 × 10^5^ cells/well) onto pre-coated Transwell chambers (polycarbonate membrane, 0.4 μm pore size). The TEER was monitored daily until stable plateau values were achieved (>150 Ω·cm^2^), indicating the formation of a confluent monolayer. TEER was measured using a Millicell ERS-2 voltohmmeter at the indicated time points and expressed as a percentage of the resistance measured in blank membrane controls (medium only).

NaFl Permeability Assay. Following TEER measurement, NaFl solution (100 μg/mL) was added to the upper chamber, while an equal volume of blank medium was placed in the lower chamber. After 1 h of incubation at 37 °C, 100-μL aliquots were collected from the lower chamber. Fluorescence intensity was measured (excitation/emission: 480/538 nm), and the apparent permeability coefficient (P_app_, cm/s) was calculated using standard curves.

### 2.12. Transcriptome Sequencing

RNA sequencing and data analysis were performed by Novogene Co., Ltd. (Shanghai, China). Total RNA was extracted from primary rBMECs treated with Hcy for 24 h using TRIzol reagent. RNA integrity and purity were assessed using an Agilent 2100 Bioanalyzer (Agilent Technologies, Santa Clara, CA, USA); samples with RNA integrity number (RIN) > 9 and A_260_/A_280_ > ratio ≈ 2.1 were selected for subsequent analysis. The mRNA was enriched from total RNA using oligo(dT) magnetic beads. Following fragmentation, first-strand cDNA was synthesized using random hexamer primers, followed by second-strand cDNA synthesis. The cDNA libraries were then prepared through end repair, A-tailing, adapter ligation, fragment size selection, PCR amplification, and purification. Library concentration was quantified using a Qubit fluorometer and real-time PCR, and fragment size distribution was assessed using a Bioanalyzer. Qualified libraries were pooled according to effective concentration and target data volume requirements, and sequenced on an Illumina platform. Differential expression analysis between the homocysteine (Hcy)-treated group and the vehicle control group was performed using the DESeq2 R package (version 1.42.0), based on gene-level count data obtained from alignment to the Rattus norvegicus reference genome assembly mRatBN7.2 (rn7). DESeq2 models count data using a negative binomial distribution and provide empirical shrinkage for dispersion and fold change estimation. To account for multiple hypothesis testing, the Benjamini–Hochberg procedure was applied to adjust raw *p*-values, thereby controlling the false discovery rate (FDR). Genes with an adjusted *p*-value ≤ 0.05 and an absolute log_2_ fold change (|log_2_FC|) ≥ 1 were considered differentially expressed.

### 2.13. Flow Cytometric Sorting of Hippocampal Endothelial Cells

Hippocampal tissues were dissected from control and CUMS rats after modeling and immediately placed in ice-cold PBS. Tissues were minced into small pieces (approximately 1 mm^3^) using sterile scissors, then digested with a mixture of collagenase type II (1 mg/mL) and DNase I (0.1 mg/mL) in Hank’s balanced salt solution at 37 °C for 30 min with gentle agitation. The digestion reaction was terminated by adding an equal volume of ice-cold PBS containing 10% FBS. The resulting cell suspension was filtered through a 70 μm cell strainer to remove undigested tissue fragments and cell aggregates, then centrifuged at 300× *g* for 5 min at 4 °C. The cell pellet was washed twice with cold PBS containing 1% FBS to remove residual enzymes.

For immunostaining, the single-cell suspension was adjusted to a concentration of 1 × 10^6^ cells per 100 μL in staining buffer (PBS with 2% FBS). Cells were then stained with FITC-conjugated anti-rat CD45 antibody (BD Biosciences, San Jose, CA, USA, 554877) and PE-conjugated anti-rat CD31 antibody (BD Biosciences, 555027) at optimized dilutions (1:100) for 30 min at 4 °C in the dark. Following antibody incubation, cells were washed twice with staining buffer and centrifuged at 300× *g* for 5 min. After the final wash, cells were resuspended in staining buffer for immediate flow cytometric sorting. Endothelial cells were identified as CD45^−^/CD31^+^ population and sorted using a flow cytometer (BD FACSAria™, San Jose, CA, USA, III) equipped with appropriate lasers and filters. Sorted cells were collected into sterile tubes containing RNase inhibitor or lysis buffer for downstream analyses as required.

Sorted endothelial cells were collected into sterile 1.5 mL microcentrifuge tubes containing 500 μL of ice-cold PBS and centrifuged at 300× *g* for 5 min at 4 °C. The supernatant was carefully aspirated, and the cell pellet was washed once with 500 μL of ice-cold PBS to remove residual staining buffer and antibodies. After centrifugation under the same conditions, the supernatant was discarded, and the cell pellet was snap-frozen in liquid nitrogen and stored at −80 °C until DNA extraction. The cell pellet was used for Microscale 5-mC Methylome Sequencing and RT-qPCR.

### 2.14. Microscale 5-mC Methylome Sequencing

For whole-genome bisulfite sequencing (WGBS), DNA extraction was performed by Novogene Co., Ltd. (Shanghai, China) using a magnetic bead-based universal genomic DNA extraction kit (Tiangen, Beijing, China, DP705). Genomic DNA degradation and contamination were assessed by agarose gel electrophoresis. DNA purity was determined using a NanoPhotometer^®^ spectrophotometer (IMPLEN, Westlake Village, CA, USA). Fragment size distribution was examined using an Agilent 2100 Bioanalyzer (Agilent Technologies, Santa Clara, CA, USA). DNA concentration was measured using the Qubit^®^ dsDNA HS Assay Kit with a Qubit^®^ 2.0 Fluorometer (Life Technologies, Carlsbad, CA, USA). Unmethylated λDNA (as a negative control) was spiked into 10–100 ng of DNA, and samples were fragmented to 200–350 bp using a Bioruptor Pico sonication device (Heidolph Instruments, Schwabach, Germany). Libraries were constructed after enzymatic treatment with TET2 and APOBEC3A. Library quality was assessed on an Agilent 2100 Bioanalyzer system. Sequencing was performed on an Illumina platform (Illumina, San Diego, CA, USA) using a paired-end 150 bp (PE150) strategy. The sequencing coverage for all six samples ranged from 18× to 24×. Raw data quality was assessed with FastQC (v0.11.3). Raw sequencing reads in FASTQ format were preprocessed using fastp (v0.20.0) to remove adapters and low-quality reads. Clean reads were aligned to the reference genome using Bismark (v0.16.3). Subsequent analyses included methylation level calling, differential methylation analysis, and Gene Ontology (GO) and Kyoto Encyclopedia of Genes and Genomes (KEGG) enrichment analysis of genes associated with differentially methylated regions (DMRs). DMRs were identified using the DSS software (version 2.50.0, Bioconductor). The final DMRs were selected based on the following criteria: an absolute difference in mean methylation levels between the two groups of ≥10%, and a *p*-value ≤ 0.05.

### 2.15. Methylation-Specific PCR (MSP)

Genomic DNA was extracted from rat hippocampal tissues using a DNA extraction kit (Vazyme, DC112) according to the manufacturer’s instructions. DNA concentration and purity were assessed using a U-drop spectrophotometer. Samples with an A_260_/A_280_ ratio between 1.8 and 2.0 were selected for subsequent bisulfite conversion.

Purified genomic DNA (500 ng–1 μg) was treated with a bisulfite conversion kit (Vazyme, EM112) following the manufacturer’s protocol. This process deaminates unmethylated cytosines to uracil, while methylated cytosines (5-mC) remain unchanged. Converted DNA was eluted and stored at −20 °C. Methylation-specific PCR (MSP) was performed to assess the methylation status of the intron region of *Cacna1c*. The initially identified Cacna1c hypomethylated intronic region from sequencing data spanned chr4:151,764,138–152,379,454 (based on the mRatBN7.2 assembly). However, due to inherent alignment discrepancies between the sequencing reads and the reference genome, we manually corrected the coordinates. The corrected fragment is located immediately downstream of the transcription start site (TSS) at chr4:153,495,067–153,495,299. This region was extracted and used for MSP primer design via the MethPrimer online tool. Primer sequences are listed in Table S2.

Each PCR reaction (20 μL) contained 2 μL of bisulfite-converted DNA, 10 μL of 2× Taq PCR Master Mix, and 0.4 μM of each primer. The amplification conditions were as follows: initial denaturation at 95 °C for 5 min; 40 cycles of 95 °C for 30 s, annealing at 60 °C for 30 s, and 72 °C for 30 s; and a final extension at 72 °C for 7 min. PCR products were separated by electrophoresis on 2% agarose gels containing GelRed nucleic acid stain in 1× TAE buffer at 100 V for approximately 40 min. Gels were visualized under UV illumination. Methylation status was determined based on the presence or absence of bands amplified by methylated versus unmethylated primer sets.

### 2.16. Statistical Analysis

Statistical analyses were performed using GraphPad Prism (version 10.1; GraphPad Software, La Jolla, CA, USA). Data are presented as mean ± standard error of the mean (SEM). Exact sample sizes, statistical tests used, and the corresponding results are provided in the figure legends. Normality was assessed using the D’Agostino–Pearson omnibus test; when the normality assumption was violated, non-parametric tests were applied. Comparisons between two groups were performed using two-tailed Student’s *t*-test. For multiple group comparisons, one-way analysis of variance (ANOVA) followed by Tukey’s post hoc test, or two-way ANOVA followed by Bonferroni’s post hoc test, was used as appropriate. Statistical significance was defined as *p* < 0.05.

## 3. Results

### 3.1. Chronic Stress Induces Hippocampus-Predominant BBB Disruption in Rats

**Figure 1:**
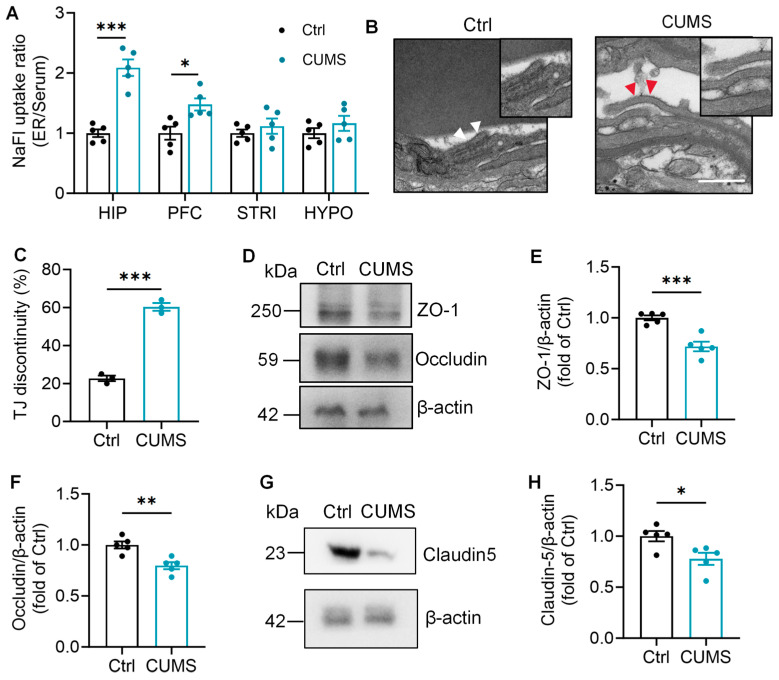
Chronic stress induces hippocampus-predominant BBB disruption in rats. (**A**) Normalized BBB permeability to NaFl in stress-related brain regions of rats (Student’s unpaired *t* test, *n* = 5). The black bar represents the “Ctrl” group, and the blue bar represents the “CUMS” group (left and right bars in each pair, respectively). (**B**) Representative TEM images of TJ structures in the hippocampus of rats, Scale bar: 500 nm. Intact TJs are indicated by white arrows, and discontinuous TJs are indicated by red arrows. (**C**) Quantification of the percentage of discontinuous tight junctions in (**B**) (Student’s unpaired *t* test, *n* = 3 rats, 20–25 TJs per rat). (**D**) Representative Western blot images showing protein levels of Occludin and ZO-1 in the hippocampus. (**E**,**F**) Quantitative analysis of Occludin and ZO-1 protein levels in (**E**) (Student’s unpaired *t* test, *n* = 5). (**G**) Representative Western blot images showing protein levels of Claudin-5 in the hippocampus. (**H**) Quantitative analysis of Claudin-5 protein levels in (**E**) (Student’s unpaired *t* test, *n* = 5). Data are expressed as mean ± SEM. * *p* < 0.05, ** *p* < 0.01, *** *p* < 0.001, vs. Ctrl.

Rats exhibited cognitive decline after 8 weeks of CUMS, including a lower recognition index in the NORT, and decreased crossing numbers and longer escape latency to target platform in the MWMT (Figure S1). To determine whether CUMS induces region-specific BBB dysfunction, we first quantified BBB permeability across four brain regions including hippocampus, prefrontal cortex (PFC), striatum and hypothalamus using the NaFl tracer method. CUMS rats exhibited a profound increase in NaFl uptake specifically within the hippocampus, along with a significant but milder increase in the PFC (Figure 1A). In contrast, NaFl extravasation in the striatum and hypothalamus remained unaffected (Figure 1A). These findings indicate that chronic stress selectively compromises BBB integrity, resulting in pathological hyperpermeability that is most pronounced in the hippocampus.

Given that functional impairment is often rooted in morphological alterations, we next examined the ultrastructural integrity of hippocampal microvessels using transmission electron microscopy (TEM). The results showed that the continuous TJs observed in controls were severely fragmented with visible gaps following CUMS exposure (Figure 1B). Quantitative analysis confirmed a significant increase in TJ discontinuity (Figure 1C).

Consistent with the structural damage, Western blotting further confirmed a disrupted BBB molecular architecture in CUMS rats, evidenced by significant reductions in ZO-1, Occludin and Claudin-5 (Figure 1D–H). Collectively, these findings demonstrate that CUMS triggers a hippocampus-predominant breakdown of the BBB, characterized by loss of both structural and functional barrier integrity.

### 3.2. VBco-Mediated Hcy Reduction Reverses Chronic Stress-Induced BBB Disruption

**Figure 2:**
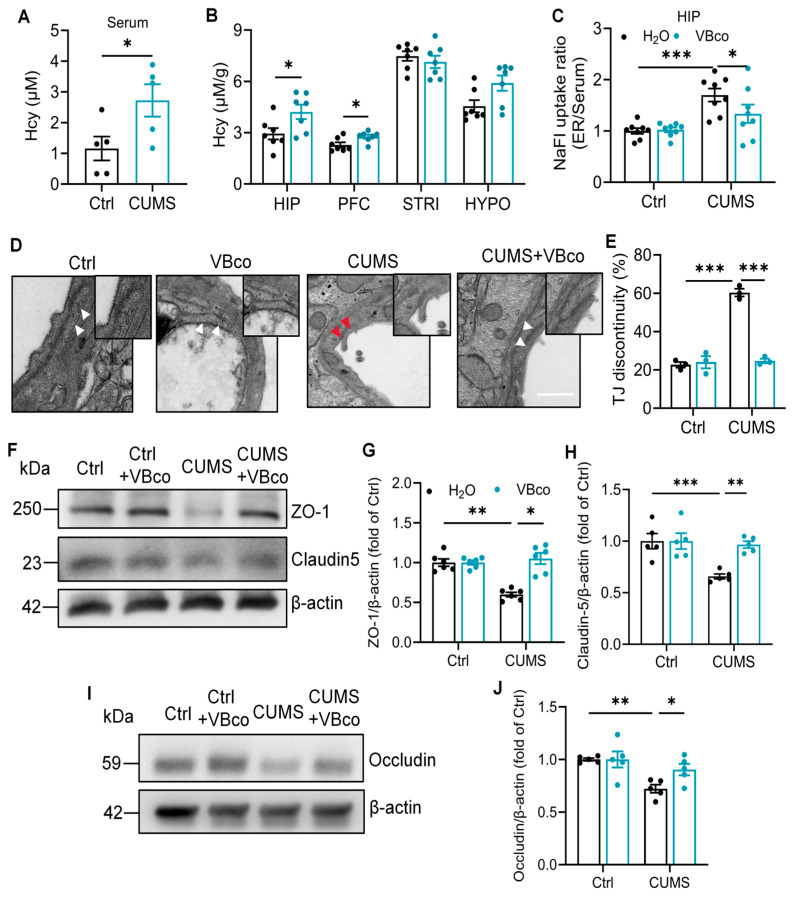
VBco-mediated Hcy reduction reverses chronic stress-induced BBB disruption. (**A**) Comparison of Hcy levels in rat serum and stress-related brain regions (Student’s unpaired *t* test, *n* = 5). (**B**) Comparison of Hcy levels in rat serum and stress-related brain regions (Student’s unpaired *t* test, *n* = 7). The black bar represents the “Ctrl” group, and the blue bar represents the “CUMS” group (left and right bars in each pair, respectively). (**C**) Normalized BBB permeability to NaFl in the hippocampus (two-way ANOVA followed by Tukey’s post hoc test, *n* = 8). (**D**) Representative TEM images of TJ structures in the hippocampus of rats, Scale bar: 500 nm. Intact TJs are indicated by white arrows, and discontinuous TJs are indicated by red arrows. (**E**) Quantification of the percentage of discontinuous tight junctions in (**D**) (two-way ANOVA followed by Tukey’s post hoc test, *n* = 3 rats, 20–25 TJs per rat). The black bar represents the group administered with saline by gavage, and the blue bar represents the group administered with VBco by gavage (left and right bars in each pair, respectively). (**F**) Representative Western blot images showing protein levels of Claudin-5 and ZO-1 in the hippocampus. (**G**,**H**) Quantitative analysis of Claudin-5 and ZO-1 protein levels in (**F**) (two-way ANOVA followed by Tukey’s post hoc test, ZO-1 *n* = 6, Claudin-5 *n* = 5). The black bar represents the group administered with saline by gavage, and the blue bar represents the group administered with VBco by gavage (left and right bars in each pair, respectively). (**I**) Representative Western blot images showing protein levels of Occludin in the hippocampus. (**J**) Quantitative analysis of Occludin protein levels in (**I**) (two-way ANOVA followed by Tukey’s post hoc test, *n* = 5). The black bar represents the group administered with saline by gavage, and the blue bar represents the group administered with VBco by gavage (left and right bars in each pair, respectively). The data are presented as the mean ± SEM. * *p* < 0.05, ** *p* < 0.01, *** *p* < 0.001, vs. Ctrl; vs. CUMS.

To establish the pathological link between chronic stress, Hcy and BBB integrity, we first profiled Hcy levels in different brain regions following CUMS exposure. The results showed that CUMS significantly elevated systemic serum Hcy (Figure 2A) as well as locally in the hippocampus and PFC, but not in the striatum or hypothalamus (Figure 2B and Figure S4). Given that the hippocampus exhibited the most severe BBB disruption alongside this significant Hcy accumulation, we focused on hippocampal tissue for the subsequent mechanistic investigations.

To determine whether Hcy accumulation is causal for BBB damage, we utilized the VBco comprising VB_6_, folic acid and VB_12_, the essential cofactors to promote Hcy metabolism, to counteract Hcy accumulation in hippocampus of CUMS mice. VBco supplementation successfully reversed CUMS-induced cognitive deficits, restoring the prolonged escape latencies and reduced platform crossings in the MWMT and increasing the recognition index in the NORT (Figure S2). The NaFl tracer assays further demonstrated that VBco treatment restored hippocampal hyperpermeability in the CUMS rats (Figure 2C). TEM assay also revealed that the TJ discontinuity caused by CUMS were remarkably reduced following VBco treatment (Figure 2D,E). In addition, compared with the untreated CUMS group, VBco upregulated the expression of the TJ proteins including Claudin-5, Occludin, and ZO-1 (Figure 2F,J). Together, these results indicate that promoting Hcy metabolism is sufficient to restore the BBB integrity under chronic stress.

### 3.3. Hcy Directly Impairs Barrier Function in Primary Rat Brain Microvascular Endothelial Cells

**Figure 3:**
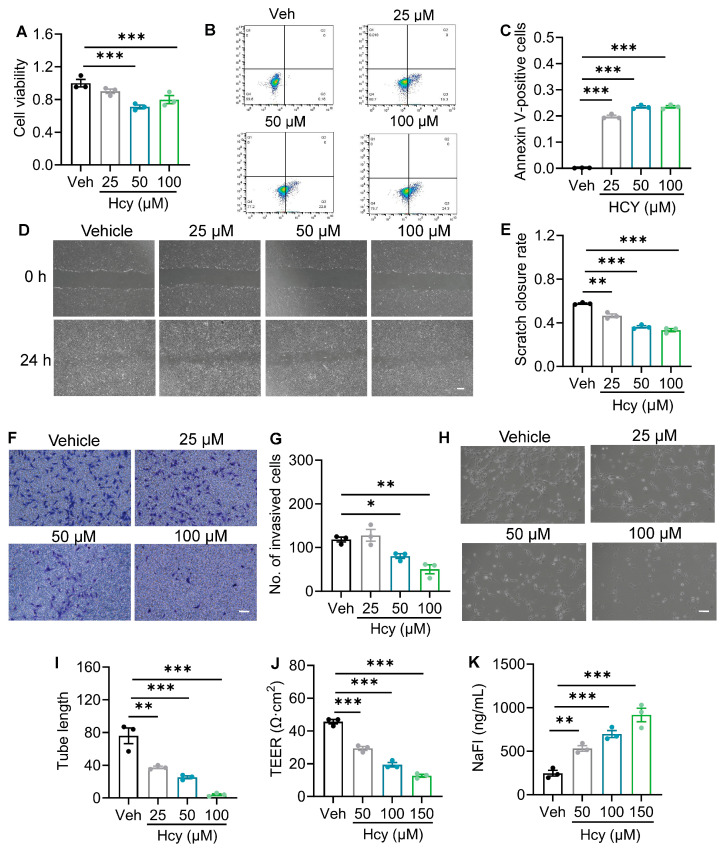
Hcy directly impairs barrier function in primary rat brain microvascular endothelial cells. (**A**) Cell proliferation of rBMECs treated with Hcy at 48 h assessed by CCK-8 assay (one-way ANOVA followed by Tukey’s post hoc test, *n* = 3). (**B**) Representative flow cytometry plots showing apoptosis in Hcy-treated rBMECs at 24 h detected by Annexin V-FITC/7-AAD double staining. (**C**) Apoptosis of Hcy-treated rBMECs at 24 h detected by 7-AAD staining (one-way ANOVA followed by Tukey’s post hoc test, *n* = 3). (**D**) Representative scratch wound healing images at 0 and 24 h. Scale bar: 200 μm. (**E**) Quantification of migration distance (one-way ANOVA followed by Tukey’s post hoc test, *n* = 3). (**F**) Representative Transwell migration images. Scale bar: 200 μm. (**G**) Quantification of migrated cells (one-way ANOVA followed by Tukey’s post hoc test, *n* = 3). (**H**) Representative tube formation images. Scale bar: 200 μm. (**I**) Quantification of tube length (one-way ANOVA followed by Tukey’s post hoc test, *n* = 3). (**J**) TEER values in Hcy-treated cells (one-way ANOVA followed by Tukey’s post hoc test, *n* = 3). (**K**) NaFl permeability in Hcy-treated cells (one-way ANOVA followed by Tukey’s post hoc test, *n* = 3). The data are presented as the mean ± SEM. * *p* < 0.05, ** *p* < 0.01, *** *p* < 0.001, vs. Veh.

To determine whether Hcy directly contributes to BBB disruption, we tested the effects of Hcy on BMEC function using rBMECs (The cell purity was verified by immunofluorescence staining, as shown in Figure S3). Our results showed that exposure to Hcy (25, 50, and 100 μM) dose-dependently suppressed rBMEC proliferation as assessed by the CCK-8 assay (Figure 3A), and significantly increased apoptotic cell death, as measured by 7-AAD staining (Figure 3B,C). Moreover, both wound-healing and Transwell migration assays indicated that Hcy delayed scratch closure (Figure 3D,E) and reduced the number of invading cells (Figure 3F,G), exhibiting the inhibitory effect on cell migration. In addition, tube formation assays demonstrated that Hcy severely disrupted angiogenic capacity, shortening the total tube length compared to the intact capillary-like networks in the control group (Figure 3H,I).

To directly evaluate the effect of Hcy on endothelial barrier integrity, we measured TEER and NaFl permeability across rBMEC monolayers. Consistent with the functional impairments observed above, Hcy treatment significantly reduced TEER values (Figure 3J) and increased paracellular NaFl leakage (Figure 3K), indicating a loss of endothelial barrier tightness. Collectively, these findings provide direct in vitro evidence that Hcy compromises BBB function by suppressing BMEC viability, migration, tube formation, and physical barrier integrity.

### 3.4. Genome-Wide Identification of Target Genes of Hcy in rBMECs

**Figure 4:**
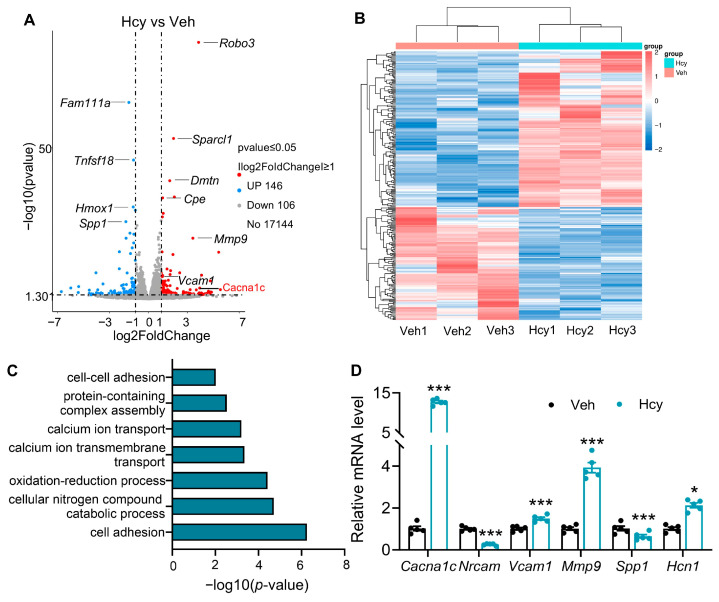
Transcriptomic profiling identifies Hcy-regulated genes in rBMECs. (**A**) Volcano plot of DEGs in rBMECs treated with Hcy. (**B**) Heatmap of DEGs in Hcy-treated rBMECs. (**C**) GO enrichment analysis of DEGs associated with BBB function or oxidative stress. (**D**) mRNA expression levels of representative DEGs validated by RT-qPCR in rBMECs treated with Hcy (Student’s unpaired *t* test, n = 5). The data are presented as the mean ± SEM. * *p* < 0.05, *** *p* < 0.001, vs. Veh.

To elucidate the molecular mechanisms by which Hcy regulates BMEC function and BBB integrity, we performed high-throughput RNA sequencing in rBMECs following Hcy treatment. Compared with vehicle-treated controls, Hcy exposure resulted in 252 differentially expressed genes (DEGs), including 146 upregulated and 106 downregulated transcripts (Figure 4A,B). GO enrichment analysis revealed that these DEGs were primarily involved in cell adhesion and proteolysis, providing further evidence that Hcy triggers the structural degradation of the endothelial barrier (Figure 4C). Notably, a significant subset of DEGs was also enriched in oxidative stress-related pathways including ROS and oxidation-reduction processes (Figure 4C), indicating the redox dysregulation induced by Hcy in BMECs.

To validate the transcriptomic findings, we performed qRT-PCR on representative DEGs involved in these enriched pathways, including *Vcam1*, *Spp1*, *Cacna1c*, *Nrcam*, *Hcn1* and *Mmp9*. The expression patterns of these genes were fully consistent with the RNA-seq results (Figure 4D). Together, these results demonstrate that Hcy induces a transcriptomic profile characterized by severe oxidative stress and structural barrier breakdown.

### 3.5. Cav1.2 Upregulation Mediates Hcy-Induced Oxidative Stress and TJ Degradation in rBMECs

**Figure 5:**
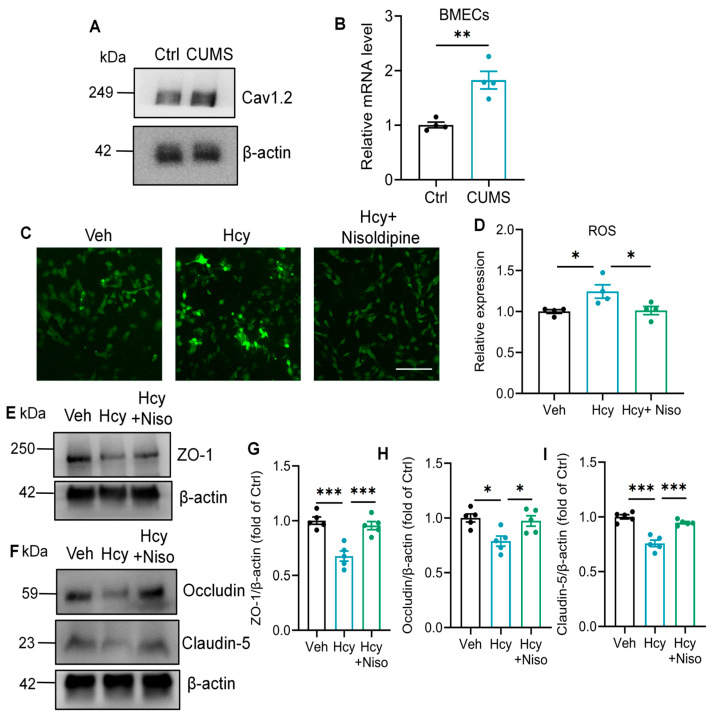
Cav1.2 upregulation mediates Hcy-induced oxidative stress and TJ degradation in rBMECs. (**A**) Representative Western blot images showing protein levels of Cacna1c in the hippocampus. (**B**) Cav1.2 mRNA expression in BMECs of CUMS rats treated with Hcy detected by qRT-PCR (Student’s unpaired *t* test, *n* = 4). (**C**) Representative fluorescent images of ROS in rBMECs treated with Hcy for 12 h. Scale bar: 200 μm. (**D**) Quantification of ROS fluorescence intensity (one-way ANOVA followed by Tukey’s post hoc test, *n* = 4). (**E**) Representative Western blot images showing protein levels of ZO-1 in the rBMECs. (**F**) Representative Western blot images showing protein levels of Occludin and Claudin-5 in the rBMECs. (**G**) Quantitative analysis of ZO-1 protein levels in (**E**) (one-way ANOVA followed by Tukey’s post hoc test, *n* = 5). (**H**,**I**) Quantitative analysis of ZO-1 protein levels in (**E**) (one-way ANOVA followed by Tukey’s post hoc test, *n* = 5). The data are presented as the mean ± SEM. * *p* < 0.05, ** *p* < 0.01, *** *p* < 0.001, vs. Veh; vs. Hcy.

It is well established that intracellular calcium overload typically driven by ion channel dysfunction is a primary upstream trigger for excessive ROS generation [18,19]. Notably, Hcy exposure induced significant expression changes in calcium ion transport (Figure 4C), among which Cav1.2 (encoded by *Cacna1c*) emerged as a prominent candidate. First, to test whether Cav1.2 serves as the molecular link between Hcy exposure and downstream endothelial dysfunction, we first validated its protein expression in rBMECs. q-PCR and Western blot analysis confirmed that Hcy significantly upregulated the mRNA and protein levels of Cav1.2 in the BMECs of CUMS rats (Figure 5A,B).

We then investigated whether Cav1.2 activation is responsible for the Hcy-induced redox imbalance. As expected, Hcy treatment triggered the ROS overproduction; however, pharmacological inhibition of Cav1.2 with nisoldipine attenuated this oxidative surge (Figure 5C,D), suggesting the role of Cav1.2 in Hcy-induced oxidative stress. Further, we assessed the impact of Cav1.2 blockade on endothelial structural integrity. The results showed that administration of nisoldipine effectively prevented the repression of Hcy on TJ proteins including Claudin-5, Occludin and ZO-1 (Figure 5E,I). Together, these findings demonstrate that the Hcy-induced upregulation of Cav1.2 acts as a significant trigger for endothelial barrier dysfunction.

### 3.6. Hcy-Induced DNA Hypomethylation Facilitates the Transcriptional Activation of Cav1.2 in rBMECs

**Figure 6:**
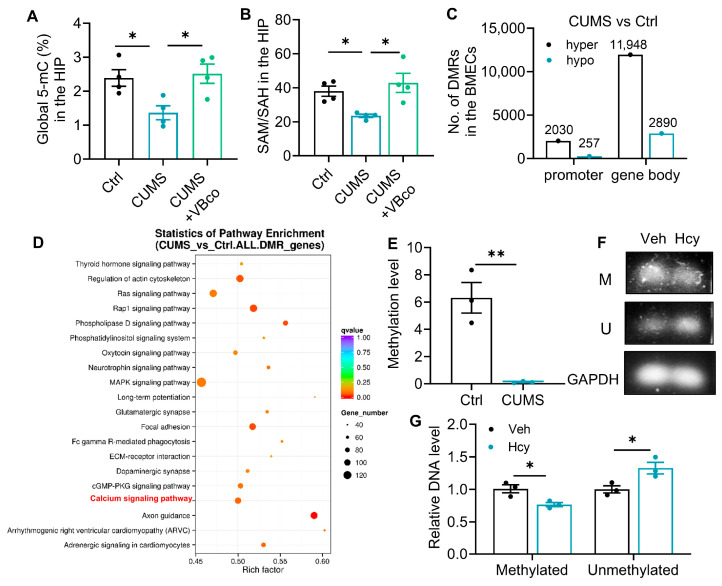
Hcy-induced DNA hypomethylation facilitates the transcriptional activation of Cav1.2 in rBMECs. (**A**) SAM/SAH ratio in the hippocampus of CUMS mice (one-way ANOVA followed by Tukey’s post hoc test, *n* = 3). (**B**) Global DNA 5-mC levels in the hippocampus of CUMS mice (one-way ANOVA followed by Tukey’s post hoc test, *n* = 3). (**C**) WGBS profiling showing DNA methylation changes in BMECs isolated from CUMS mice. (**D**) GO pathway enrichment analysis of DMRs identified by WGBS. The Cav1.2-related pathway is highlighted in red. (**E**) Quantification of DNA methylation levels at hypomethylated sites within the Cacna1c gene identified by WGBS (Student’s unpaired *t* test, *n* = 3). (**F**) Representative MSP images showing DNA methylation levels within the intronic region of Cav1.2 in rBMECs treated with Hcy. (**G**) Quantification of DNA 5-mC levels in the Cav1.2 intronic region assessed by MSP (one-way ANOVA followed by Tukey’s post hoc test, *n* = 3). The data are presented as the mean ± SEM. * *p* < 0.05, ** *p* < 0.01, vs. Ctrl; vs. CUMS; vs. Veh; vs. Hcy.

Given that Hcy is a critical intermediate in one-carbon metabolism and a well-known disruptor of DNA methylation, we further investigated whether the upregulation of Cav1.2 is regulated through epigenetic modifications. We observed a significant reduction in the SAM/SAH ratio within the hippocampus of CUMS mice, accompanied by a decline in global DNA 5-mC levels (Figure 6A,B), suggesting a state of systemic hypomethylation. Subsequent WGBS profiling of isolated BMECs confirmed extensive epigenetic remodeling induced by CUMS (Figure 6C). While stress induced widespread DNA hypermethylation across many loci (2030 in promoters, 11,948 in gene bodies), a crucial subset of regions exhibited targeted hypomethylation (257 in promoters, 2890 in gene bodies), aligning with the systemic SAM/SAH deficit. Additionally, these DMRs highlighted genes involved in calcium signaling pathway, focal adhesion and regulation of actin cytoskeleton (Figure 6D).

Notably, Cav1.2 was identified as a key candidate among the differentially methylated genes, and there are significant hypomethylated sites within its intron (Figure 6E). We further validated the methylation status of Cav1.2 in rBMECs following Hcy treatment. MSP targeting the identified DMRs revealed that Hcy exposure significantly decreased the DNA 5-mC levels within an intronic region of the Cav1.2 gene (Figure 6F,G). These results suggest that Hcy-driven depletion of methyl donors is associated with intronic hypomethylation of Cav1.2, which may contribute to its upregulated expression in BMECs.

## 4. Discussion

In the present study, we identify Hcy as a critical driver in CUMS-induced BBB dysfunction and cognitive impairment. We demonstrated that CUMS selectively compromises BBB integrity in the hippocampus, a region vital for learning and memory. Lowering Hcy levels with VBco supplementation restored the hippocampal barrier and alleviated cognitive deficits. Mechanistically, Hcy disrupted BBB integrity by inducing DNA hypomethylation-mediated derepression of Cav1.2, which in turn overproduced ROS that impaired endothelial function. Overall, these findings identify Hcy-driven epigenetic dysregulation as a promising therapeutic target for mitigating neurovascular impairment under chronic stress.

Our finding that chronic stress selectively targets the hippocampal BBB aligns with previous reports showing increased permeability and tight junction (TJ) downregulation in this region following CUMS or foot-shock stress [20,21]. The preferential vulnerability of the hippocampus to BBB damage likely arises from the convergence of its high glucocorticoid receptor (GR) expression density, heavy metabolic and oxidative stress burden, and distinct astrocyte–pericyte interaction patterns [22,23]. Chronic stress drives sustained glucocorticoid elevation via HPA axis hyperactivation, which preferentially targets GR-rich hippocampal regions to directly downregulate tight junction protein expression and induce mitochondrial ROS production. Concurrently, the heightened reactivity of hippocampal astrocytes to inflammatory signals further amplifies the release of matrix metalloproteinases and pro-inflammatory cytokines, thereby disrupting neurovascular unit homeostasis. However, BBB disruption is not exclusively limited to the hippocampus. Other paradigms, such as restraint or chronic social defeat stress, have been shown to induce microvascular damage and Claudin-5 loss in the PFC and nucleus accumbens [24,25]. Taken together, these discrepancies suggest that the regional vulnerability of the BBB is highly model-dependent. Nonetheless, how distinct stressors dictate these spatial patterns of neurovascular injury remains a compelling question for future research.

The critical role of Hcy in cognitive and cerebrovascular disorders has been extensively documented [13,26]. Importantly, BBB integrity was damaged in models of HHcy, leading to increased cortical permeability and downregulation of TJ proteins [27,28]. Consistent with these findings, we observed significant BBB breakdown in the hippocampus of CUMS rats, which coincided with a marked elevation of local Hcy levels. Also, we suggested that Hcy at mildly to moderately elevated concentrations is sufficient to impair barrier-forming functions of BMECs. Interestingly, a key observation in our study is the significant elevation of central Hcy levels in the hippocampus of CUMS rats. The origin of this localized accumulation remains debated. Some evidence suggests that chronic stress impairs local Hcy catabolism in the hippocampus and PFC, leading to accumulation in these brain regions [29]. Alternatively, elevated circulating Hcy might infiltrate the brain through an already compromised BBB [13,30]. These findings reinforce the central role of Hcy in cerebrovascular and cognitive pathology.

While studies have established Hcy as a potent disruptor of BBB integrity, the precise mechanisms, particularly in the context of chronic stress, remain incompletely understood. Previous work has delineated multiple molecular pathways driving Hcy-induced endothelial dysfunction and BBB breakdown, including reduced nitric oxide (NO) bioavailability [31], excessive oxidative stress [32], impaired hydrogen sulfide signaling coupled with NF-κB-driven inflammatory activation [33] and protein N-homocysteinylation-mediated TJ protein downregulation [34]. Consistently, our transcriptomic profiling of rBMECs exposed to Hcy showed that Hcy treatment upregulated genes involved in the extracellular matrix remodeling, inflammation, oxidative stress and proteolysis, alongside downregulation of genes associated with cell–cell junctions and protein folding, suggesting that Hcy reprograms endothelial functions at the gene expression level.

Notably, emerging evidence has linked Hcy to methylation dysregulation. Hcy accumulation leads to the buildup of SAH, a potent inhibitor of cellular methyltransferases, which has been reported to induce global DNA hypomethylation and pro-inflammatory activation in vascular endothelium [35]. For example, Hcy can upregulate p66SHC expression through promoter hypomethylation, subsequently mediating endothelial oxidative stress and inflammatory responses [36]. In our study, we revealed that stress-induced elevated Hcy suppressed DNA 5-mC modifications at the *Cacna1c* intron region to relieve transcriptional repression and consequently led to significantly elevate Cav1.2 expression. While Cav1.2 is classically associated with neuronal signaling, its aberrant expression is increasingly recognized as a driver of endothelial pathology [18]. We demonstrate that Hcy-driven Cav1.2 upregulation induced severe oxidative stress and the subsequent TJ protein degradation that disrupt the BBB.

Several limitations of the present study should be acknowledged. First, although we observed an association between Hcy-driven methyl donor depletion and intronic hypomethylation of Cav1.2, direct causal evidence demonstrating that this epigenetic alteration leads to transcriptional activation of Cacna1c remains to be established. We will employ reporter assays and chromatin immunoprecipitation analyses to further clarify the causal relationship in future studies. Additionally, while vitamin B complex supplementation effectively lowered plasma Hcy levels and partially ameliorated BBB disruption in our chronic stress model, we recognize that this intervention lacks strict specificity for Hcy metabolism. Beyond Hcy reduction, B vitamins (particularly folate, B12, and B6) participate extensively in one-carbon metabolism, which supports diverse physiological processes including DNA methylation, nucleotide synthesis, and phospholipid metabolism [37]. Consequently, the observed protective effects may partially reflect improved methylation capacity, enhanced endothelial function [38], or modulation of neurotransmitter synthesis [39] independent of Hcy lowering. Additionally this combined supplementation cannot distinguish which specific component or formulation exerts the protective effect, limiting further identification of the active ingredient(s). In the future, we will explore more specific homocysteine-regulating agents and employ gene-editing strategies targeting key Hcy-metabolizing enzymes, thereby circumventing the off-target effects inherent to vitamin B complex supplementation. We will further perform conditional Cacna1c knockout and targeted epigenetic editing of its promoter to clarify whether Hcy exerts its effects specifically through Cacna1c and how its hypomethylation contributes to this process. Finally, a limitation of this study is insufficient sample size in some in vitro experiments. Although the data show consistent and biologically meaningful differences, and are supported by in vivo evidence, we acknowledge that larger sample sizes would increase statistical power. Future studies should include more biological replicates to confirm these findings.

Future research will map the specific differentially methylated positions (DMPs) within the *Cacna1c* locus and identify the corresponding transcription factors that mediate its transcriptional activation. Additionally, the potency of *Cacna1c* methylation signatures serving as a biomarker for chronic stress-related BBB dysfunction and cognitive decline will be another focus of our future studies.

## 5. Conclusions

In conclusion, we demonstrate that chronic stress induces a region-specific BBB disruption predominantly in the hippocampus, critically driven by local Hcy accumulation. Elevated Hcy severely impairs the barrier-forming function of BMECs by triggering a Cav1.2-dependent ROS burst and TJ degradation. Importantly, lowering Hcy via vitamin B supplementation effectively restores BBB integrity and mitigates cognitive deficits. Overall, our study unveils a critical epigenetic link whereby stress-induced Hcy accumulation drives hippocampal BBB dysregulation through Cacna1c hypomethylation and subsequent Cav1.2 overexpression, providing a novel therapeutic target to preserve neurovascular health and cognitive ability against chronic stress.

## Data Availability

The transcriptomic and DNA methylomic sequencing data reported in this paper have been deposited in the Gene Expression Omnibus (GEO) database (transcriptome: GSE327052; methylation: GSE327662). These records are scheduled for public release on 16 June 2026, consistent with the journal’s data sharing policy. The original contributions presented in this study are included in the article. Further inquiries can be directed to the corresponding author.

## Supplementary Materials

The following supporting information can be downloaded at: https://www.mdpi.com/article/10.3390/brainsci16050491/s1, Figure S1: CUMS induces cognitive impairment in rats; Figure S2: VBco-mediated Hcy reduction alleviates CUMS-induced cognitive impairment in rats; Figure S3: Identification of primary rBMECs purity by CD31 immunofluorescence staining; Figure S4: VBco-mediated Hcy reduction reverses chronic stress-induced BBB disruption in different brain regions; Table S1: qRT-PCR primers; Table S2: MSP primers; Table S3: List of differentially expressed genes identified by RNA-seq.
