# Supplementary material for: Homocysteine Drives Hippocampal Blood–Brain Barrier Disruption and Cognitive Decline Under Chronic Stress via DNA Hypomethylation of Cav1.2
Source: Brain Sci. 2026 Apr 30;16(5):491. doi: 10.3390/brainsci16050491 (PMC13204877; doi:10.3390/brainsci16050491)
Supplement: Supplementary file 1 [file brainsci-16-00491-s001.zip › Supplementary information S1.pdf]

Homocysteine Drives Hippocampal Blood–Brain Barrier Disruption and Cognitive Decline Under Chronic Stress via DNA Hypomethylation of Cav1.2

Mao-Yang Zhou, Jin-Shan Li, Zhao-Xin Sun, Jie Yin, Yun Zhao, Fang Xie, Xue Wang, Sheng-Hui Zhang, Zhao-Wei Sun and Ling-Jia Qian *

Department of Neurobiology, Beijing Institute of Basic Medical Sciences, Beijing 100850, China; z18355454174@126.com (M.-Y.Z.); lj_shan99@163.com (J.-S.L.); 202110200019@stu.tjus.edu.cn (Z.-X.S.); bz3209086@126.com (J.Y.); zhaoyun@bmi.ac.cn (Y.Z.); xiefang@bmi.ac.cn (F.X.); wangxue@bmi.ac.cn (X.W.); 1701111498@pku.edu.cn (S.-H.Z.); sunzhaowei@bmi.ac.cn (Z.-W.S.)

* Correspondence: stressqian@163.com

.

Supplementary Figures

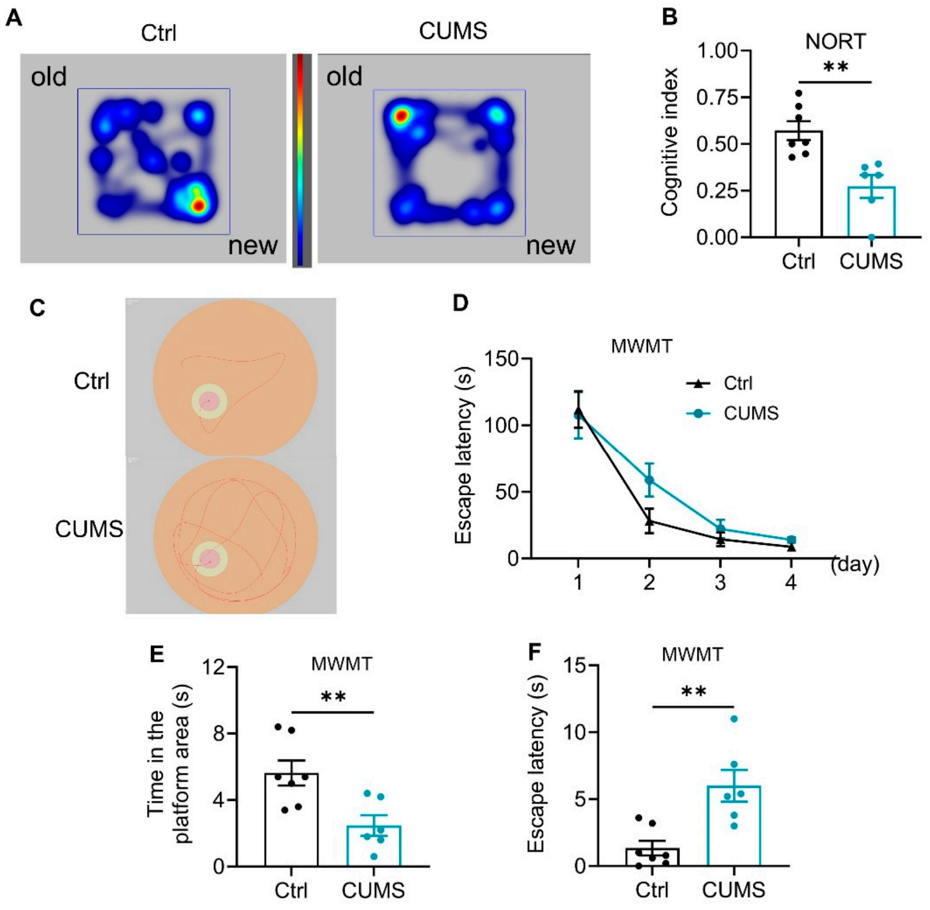

Figure S1. CUMS induces cognitive impairment in rats. (**A**) Representative heatmaps of rat locomotor trajectories in the NORT. The familiar object was placed in the upper-left corner, and the novel object was placed in the lower-right corner; red indicates more frequent visits. (**B**) Comparison of the recognition index in the NORT, n = 7. (**C**) Representative swim trajectories of rats in the MWMT. The platform area is located in the lower-left quadrant. (**D**) Line graph showing escape latency during the training phase in the MWMT. (**E**) Comparison of time spent in the platform quadrant during the test phase in the MWMT, n = 7. (**F**) Comparison of the first platform escape latency during the test phase in the MWM, n = 7. The data are presented as the mean + SEM. ** $p < 0.01$, vs. Ctrl.

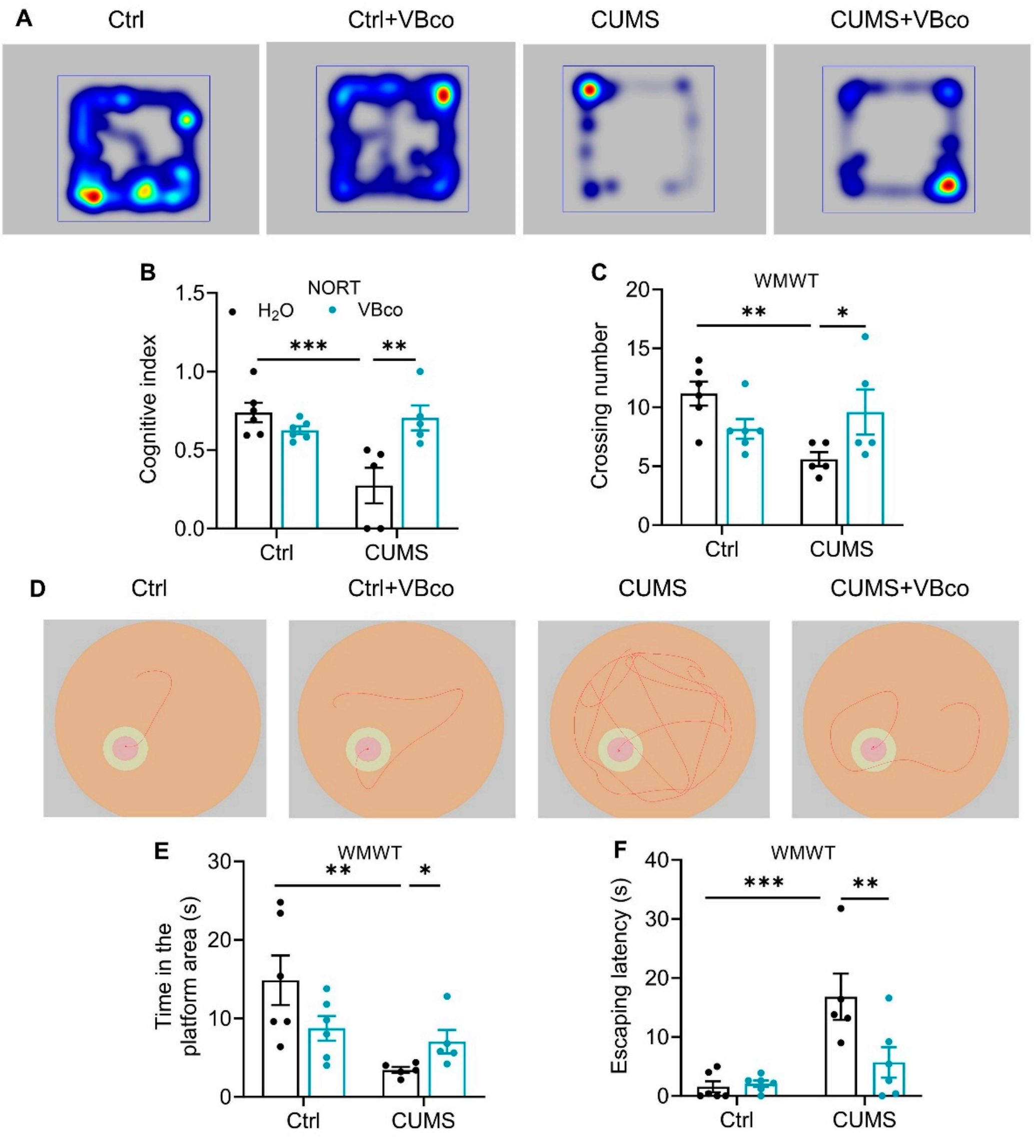

Figure S2. VBco-mediated Hcy reduction alleviates CUMS-induced cognitive impairment in rats. (**A**) Representative heatmaps of rat locomotor trajectories in the NORT. The familiar object was placed in the upper-left corner, and the novel object was placed in the lower-right corner; red indicates more frequent visits. (**B**) Comparison of the recognition index in the novel object recognition test (Two-way ANOVA followed by Tukey's post hoctest, n = 7). (**C**) Comparison of time spent in the platform quadrant during the training phase in the MWMT. The platform area islocated in the lower-left quadrant (Two-way ANOVA followed byTukey's post hoc test, n = 7). (**D**) Representative swim trajectories of rats in the MWMT. (**E**) Comparison of time spent in the platform quadrant during the test phase in the MWMT (Two-way ANOVA followed by Tukey's post hoc test, n = 7). (**F**) Comparison of the first platform crossing latency during the test phase in the MWMT (Two-way ANOVA followed by Tukey's post hoc test, n = 7). The data are presented as the mean + SEM. * $p < 0.05$, ** $p < 0.01$, *** $p < 0.001$, vs. Ctrl.

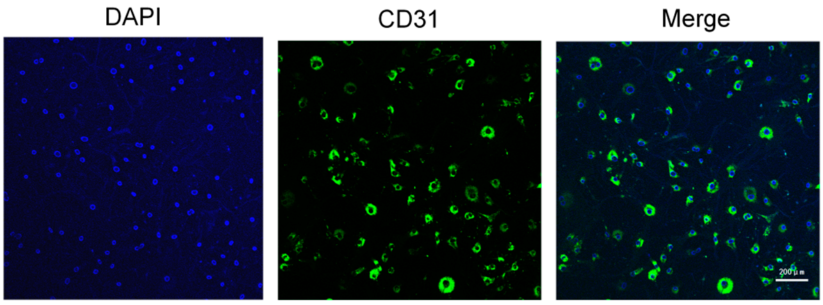

Figure S3. Identification of primary rBMECs purity by CD31 immunofluorescence staining. Representative immunofluorescence images of primary rBMECs. Nuclei were stained with DAPI (blue), CD31 (endothelial cell marker) was stained with Alexa Fluor 488-conjugated antibody (green), and merged images show co-localization. Scale bar: 200 nm.

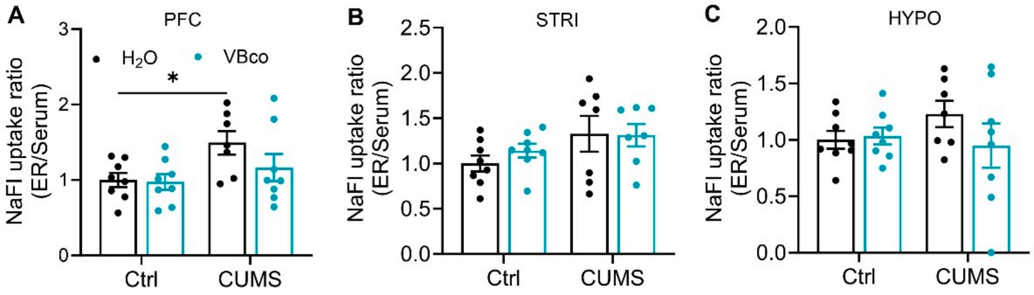

Figure S4. VBco-mediated Hcy reduction reverses chronic stress-induced BBB disruption in different brain regions. (**A**) Normalized BBB permeability to NaFl in the PFC (Two-way ANOVA followed by Tukey's post hoc test, n = 8). (**B**) Normalized BBB permeability to NaFl in the STRI (Two-way ANOVA followed by Tukey's post hoc test, n = 8). (**C**) Normalized BBB permeability to NaFl in the HYPO (Two-way ANOVA followed by Tukey's post hoc test, n = 8). The data are presented as the mean + SEM. * $p < 0.05$, vs. Ctrl+H_2O; vs. CUMS+H_2O.

Supplementary Tables

Table S1. qRT-PCR primers.

Gene	Strand	Primer (5′→3′)
Cacna1c	Forward	ATGGGCATCTTTGCGTCACA
Cacna1c	Reverse	CAAACCCGAACGGGATCTACAG
Nrcam	Forward	GGCAGCAAAGAAGAATGGAG
Nrcam	Reverse	CTTGGGTCGCAATATCCACT
Vcam1	Forward	AGTTACACAGCAGTCAAATGG
Vcam1	Reverse	CTTTCGGAGCAACGTTGAC
Mmp9	Forward	CATGTATCACTACCACGAGGA
Mmp9	Reverse	TTAGAGCCACGACCATACAG
Hcn1	Forward	AAATTCTCAGTCTCTTGCGT
Hcn1	Reverse	TCATGTGGAATATCTCTTCCC
Spp1	Forward	TTTCTGATGAACAGTATCCCGA
Spp1	Reverse	TGATAGCCTCATCGGACTC
GAPDH	Forward	AACTCCCATTCTTCCACCT
GAPDH	Reverse	TTGTCATACCAGGAAATGAGC

Table S2. MSP primers.

Primer Name	Strand	Primer (5′→3′)
Cacna1c-M	Forward	TTTTTTTTATCGTTTTTATTTTCGA
Cacna1c-M	Reverse	CTACCTACCTATACTTCCTCACGAC
Cacna1c-U	Forward	GTTTTTTTTATTGTTTTTATTTTTGA
Cacna1c-U	Reverse	CTACCTACCTATACTTCCTCACAAC

Table S3. List of differentially expressed genes identified by RNA-seq.

See Supplementary Information S2
